# A Time Series of Water Column Distributions and Sinking Particle Flux of *Pseudo-Nitzschia* and Domoic Acid in the Santa Barbara Basin, California

**DOI:** 10.3390/toxins10110480

**Published:** 2018-11-17

**Authors:** Blaire P. Umhau, Claudia R. Benitez-Nelson, Clarissa R. Anderson, Kelly McCabe, Christopher Burrell

**Affiliations:** 1 School of the Earth, Ocean & Environment, University of South Carolina, Columbia, SC 29208, USA: bumhau@email.sc.edu (B.P.U.); clrander@ucsc.edu (C.R.A.); mccabekm@email.sc.edu (K.M.); ctb0486@yahoo.com (C.B.); 2 Southern California Coastal Ocean Observing System, Scripps Institution of Oceanography, 8880 Biological Grade, La Jolla, CA 92093, USA

**Keywords:** Dissolved and particulate domoic acid, harmful algal blooms, Santa Barbara Channel, bloom toxicity

## Abstract

Water column bulk *Pseudo-nitzschia* abundance and the dissolved and particulate domoic acid (DA) concentrations were measured in the Santa Barbara Basin (SBB), California from 2009–2013 and compared to bulk *Pseudo-nitzschia* cell abundance and DA concentrations and fluxes in sediment traps moored at 147 m and 509 m. *Pseudo-nitzschia* abundance throughout the study period was spatially and temporally heterogeneous (<200 cells L^−1^ to 3.8 × 10^6^ cells L^−1^, avg. 2 × 10^5^ ± 5 × 10^5^ cells L^−1^) and did not correspond with upwelling conditions or the total DA (tDA) concentration, which was also spatially and temporally diverse (<1.3 ng L^−1^ to 2.2 × 10^5^ ng L^−1^, avg. 7.8 × 10^3^ ± 2.2 × 10^4^ ng L^−1^). We hypothesize that the toxicity is likely driven in part by specific *Pseudo-nitzschia* species as well as bloom stage. Dissolved (dDA) and particulate (pDA) DA were significantly and positively correlated (*p* < 0.01) and both comprised major components of the total DA pool (pDA = 57 ± 35%, and dDA = 42 ± 35%) with substantial water column concentrations (>1000 cells L^−1^ and tDA = 200 ng L^−1^) measured as deep as 150 m. Our results highlight that dDA should not be ignored when examining bloom toxicity. Although water column abundance and pDA concentrations were poorly correlated with sediment trap *Pseudo-nitzschia* abundance and fluxes, DA toxicity is likely associated with senescent blooms that rapidly sink to the seafloor, adding another potential source of DA to benthic organisms.

## 1. Introduction

The genus *Pseudo-nitzschia* is a marine diatom found worldwide [1], with 26 of the 49 known species capable of producing a potent neurotoxin, domoic acid (DA). Domoic acid contamination is caused predominantly by trophic transfer; particulate material (e.g., diatoms) containing DA is consumed by zooplankton and fish or ingested by filter feeding shellfish, which are subsequently consumed by marine mammals, seabirds, and humans [1,2,3,4]. Toxin-producing blooms of *Pseudo-nitzschia* have been implicated in widespread marine mammal stranding and mortality, as well as shellfish bed and beach closures [5,6,7,8,9]. Domoic acid toxicity, also known as amnesic shellfish poisoning (ASP) in humans can cause headaches, nausea, seizures, short-term memory loss, and, in more severe cases, coma and death [10,11,12]. While toxic *Pseudo-nitzschia* spp. form blooms in both open and coastal settings, they are particularly prominent during and immediately following coastal upwelling events when cold nutrient-rich waters enter the euphotic zone [1,13]. Identifying a common set of conditions that promote these blooms across regions and seasons, however, remains elusive [1]. Indeed, DA production varies greatly within and between blooms and appears to be influenced by a variety of additional environmental conditions including pH, light intensity, and salinity [12,14], relative nutrient (nitrogen, phosphorus, and silicic acid) availability and composition [15,16,17,18,19], trace metal abundance [20], and even carbon dioxide concentration [21]. Other factors include the presence of zooplankton [22,23], the composition of the bacterial community [24,25], the specific *Pseudo-nitzschia* spp. present [1] and population growth phase [26,27].

Once produced, DA may persist in the environment for days to months depending on the food web ecology, particulate versus dissolved form, and water column location (i.e., euphotic zone versus deep waters or sediments) [28,29,30]. As DA has been hypothesized to be produced predominantly in the upper water column and photodegradation is considered an important degradation pathway [28], many studies have focused on targeting marine biota and sampling surface waters, rarely sampling below 10 m [1].

It is now recognized that significant particulate DA (pDA) is produced throughout the upper water column, with particle-laden DA sinking to waters as deep as 800 m [7,17,30,31]. For example, Sekula-Wood et al. [30,32] found high quantities of DA in sediment traps at a depth of 540 m in the Santa Barbara Basin, (>20,000 ng/g sediment), thereby providing a source of DA to benthic and pelagic feeders. The transport of DA to sub-surface waters is likely mediated by secondary processes, such as fecal pellet production, marine snow aggregation, and adsorption onto the surfaces of particles [27,32,33,34] that sink rapidly, exceeding 100 m d^−1^ [30,32,35]. These particle transport processes help to explain the presence of DA in benthic food webs in the absence of an ongoing surface bloom [36,37], thereby allowing for the possibility of DA poisoning long after a toxic *Pseudo-nitzschia* bloom subsides [30,31].

The focus on pDA has also resulted in a relative paucity of the dissolved DA (dDA) measurements, which may also impact marine biota. For example, Liu et al. [38] found that dDA exposure reduced the survival and growth of larval king sea scallops, and Bargu et al. [39] showed that increasing levels of dDA suppressed the grazing rates of krill. In zebrafish, dDA exposure has been shown to cause irregular cardiovascular development [40]. More recently Van Meerssche and Pinckney [41] found that at increased salinities, the concentration of dDA inhibited the growth of certain estuarine phytoplankton species, such as cryptophytes. Other studies suggest that dDA may provide the *Pseudo-nitzschia* spp. with a competitive advantage over other organisms in low iron environments, although the mechanism is not well understood [42,43]. Combined, studies of dDA and pDA suggest that there are multiple pathways and timescales for DA to enter and impact the marine food web.

As mentioned earlier, many of the conditions hypothesized to facilitate toxic *Pseudo-nitzschia* blooms are specifically associated with coastal upwelling, including off the coasts of Southern Africa, Western Europe, South America, and the United States [1,7,44,45,46,47]. The coast of southern California, in particular, experiences regular outbreaks of toxic *Pseudo-nitzschia* blooms, with a majority of them linked to either seasonal upwelling [1,15,48,49], regional circulation (e.g., mesoscale eddies; [15]), and/or larger scale circulation patterns linked to climate [9,46]. Recent studies further suggest that there has been an increase in the frequency of southern California toxic *Pseudo-nitzschia* blooms, specifically in the Santa Barbara Basin (SBB) over the past decade and perhaps the last century [32,50]. The SBB is a hotspot for toxic *Pseudo-nitzschia* and numerous studies have identified links between seasonal upwelling conditions, *Pseudo-nitzschia* growth, and DA production in the basin [3,7,16,51,52].

This study examined the partitioning of DA between dissolved and particulate phases relative to the abundance of bulk *Pseudo-nitzschia* throughout the upper 150 m of the water column and especially with regards to upwelling, as has been shown with previous work [3,15,45]. The timing and magnitude of toxic *Pseudo-nitzschia* blooms and the downward transport of *Pseudo-nitzschia* and DA from the surface ocean to depth in the water column were also explored. Results are placed in the context of current knowledge regarding toxic *Pseudo-nitzschia* blooms not only along the California coast, but worldwide.

**Figure 1:**
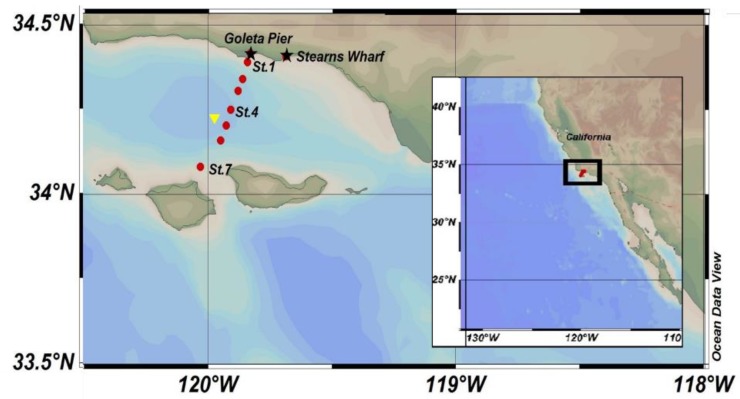
The sampling locations in the Santa Barbara Basin, California, USA. Black stars indicate pier stations. Red circles indicate offshore transect stations (1–7), and the yellow inverted triangle denotes the location of the sediment traps deployed close in proximity to Station 4.

The SBB, located off the coast of southern California (Figure 1), is a region of high primary production and particle export [53,54]. Phytoplankton blooms, dominated by diatoms, generally occur in the spring and summer in response to wind-driven upwelling and mesoscale eddies [15,51,55]. The SBB is located at the confluence of two different water masses: the surface equatorward flowing California Current (CC) that originates in the subarctic Pacific, and the poleward deeper (200–300 m) flowing California Undercurrent (CUC) that forms in the tropical northeastern Pacific [56,57,58]. These two currents are comprised of different nutrient concentrations and ratios. Changes in the relative magnitude and nutrient composition of the CC and CUC flow into the SBB have therefore been argued to play a role in the increase of toxic *Pseudo-nitzschia* blooms over the past two decades [59].

## 2. Results

### 2.1. Water Column

**Figure 2:**
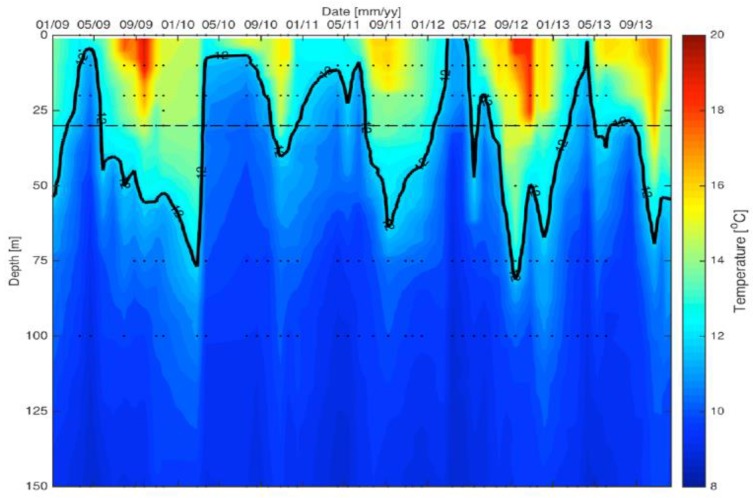
The temperature-depth profiles over the upper 150 m at Station 4 collected from 2009–2013 by the Plumes and Blooms Program. The black contour line indicates the 12 °C isotherm, which was used to define upwelling when it shoaled above 30 m, as depicted by the dashed line. Water column sample timing and depths are depicted by the black circles. All data are available in Table S1.

In order to examine the role of upwelling on *Pseudo-nitzschia* abundance and DA toxicity, upwelling events were defined as the shoaling of the 12 °C isotherm above 30 m at Station 4 (Figure 2). During the study period, the mixed layer depth ranged from 5 m to 60 m. Note that the specific mechanisms that induce upwelling, e.g., wind direction and strength as well as mesoscale eddies, require a more detailed analysis of basin physics, such as cross-shelf transport and eddy strength [60]. These analyses are beyond the scope of the present study.

#### 2.1.1. Surface Waters

**Figure 3:**
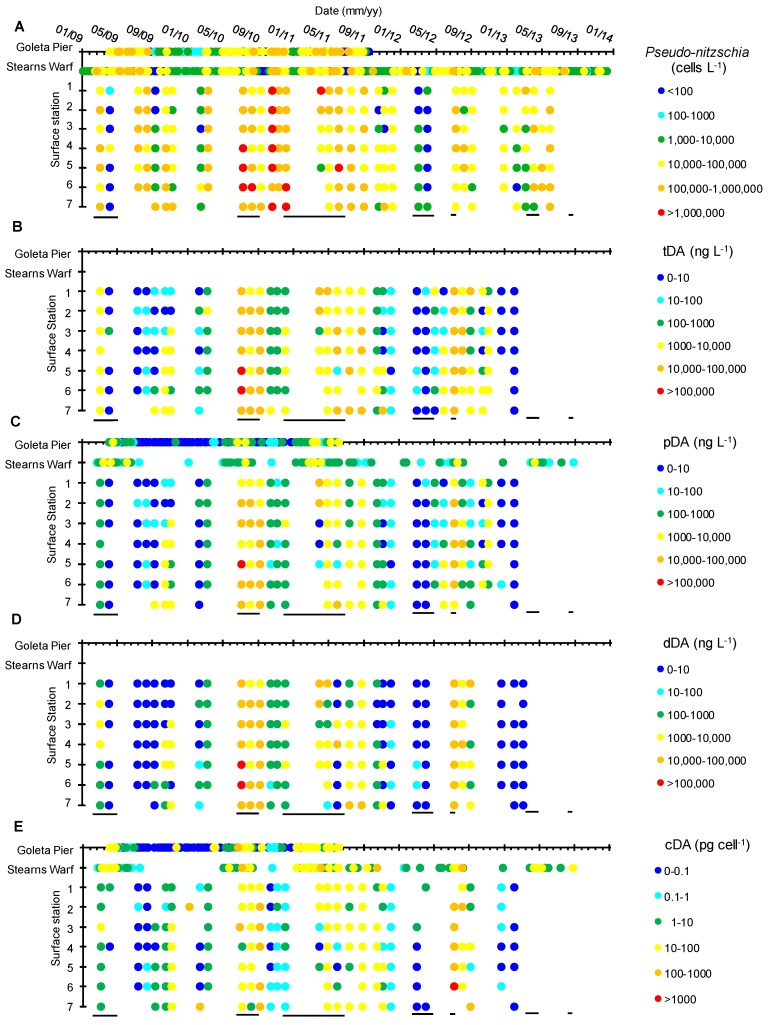
The bulk *Pseudo-nitzschia* abundance (cells L^−1^) (**A**) total domoic acid (tDA) concentrations (ng L^−1^) (**B**) particulate DA (pDA) concentrations (ng L^−1^) (**C**) dissolved DA (dDA) concentrations (ng L^−1^) (**D**), and cellular DA(cDA) concentrations (pg cell^−1^) (**E**) at all pier and surface stations (1–7 at 0 m) from 2009–2013. Upwelling periods (12 °C isotherm shoaled above 30 m) are identified by dark lines located underneath each panel. All data are available in Tables S1–S3.

**Figure 4:**
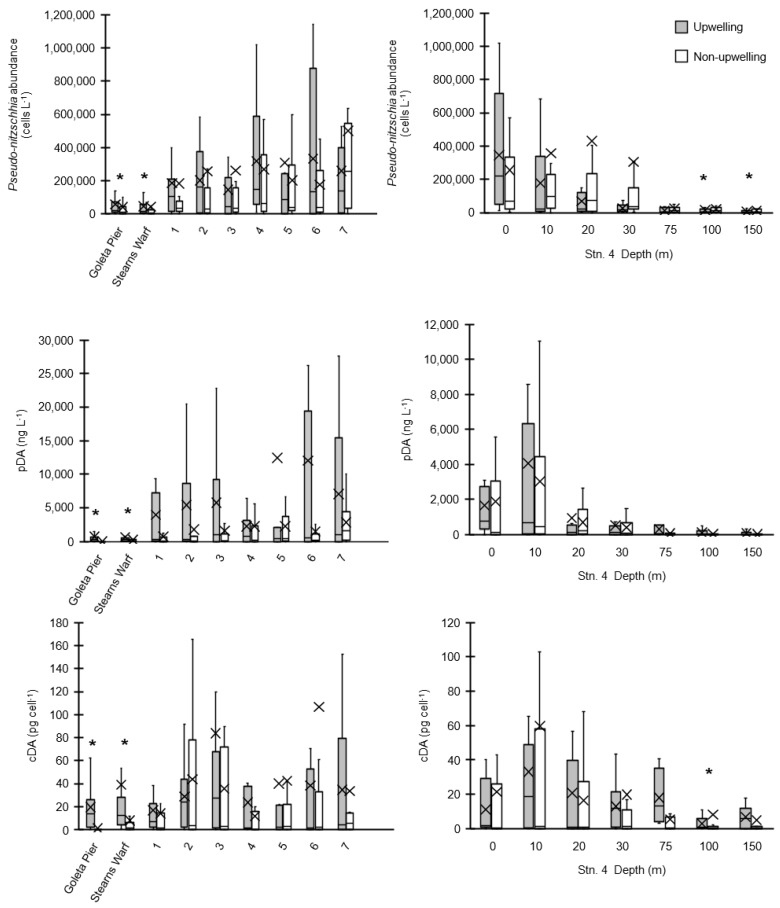
The box and whisker plots of all data collected at the pier, surface stations (1–7 at 0 m), and with depth at Station 4 (0–150 m, *n* = 7) from 2009–2013 during the upwelling (shaded bars) and non-upwelling periods (open bars) as defined by the shoaling of the 12 °C isotherm above 30 m) for bulk *Pseudo-nitzschia* abundance (cells L^−1^) (**A**,**D**), particulate domoic acid (pDA) concentrations (ng L^−1^) (**B**,**E**), and cellular DA (cDA) concentrations (pg cell^−1^) (**C**,**F**). Surface station measurements are at a depth of 0 m. In each box and whisker plot, the X denotes the average, the horizontal bar indicates the median, the box represents the 2nd and 3rd quartiles, and the vertical bars indicate the 1st (lower) and 4th (upper) quartiles. Outliers with values greater than 1.5× the interquartile range are not shown. For the full range of values, see Tables S2 and S3. The asterisk (*) above each upwelling and non-upwelling box and whisker pair indicates a significant difference (Kruskal–Wallis, *p* < 0.05).

Bulk surface *Pseudo-nitzschia* cell abundances (<5 m) along the SBB transect and at two shore-based sampling locations—Stearns Wharf and Goleta Pier (http://www.habmap.info/data.html, Figure 1)—were highly variable, with concentrations ranging from below detection (200 cells L^−1^) to >3 × 10^6^ cells L^−1^ (average 2.5 × 10^5^ ± 4.9 × 10^5^ cells L^−1^) across the SBB and from below detection to 8 × 10^5^ cells L^−1^ (average 4.1 × 10^4^ ± 9.0 × 10^4^ cells L^−1^) at the piers (Table 1, Figure 3A). While the timing of higher bulk *Pseudo-nitzschia* cell abundances measured offshore were reflected in the pier measurements, absolute concentrations often differed by an order of magnitude. Adjacent SBB transect stations often differed by more than an order of magnitude in *Pseudo-nitzschia* cell abundance as well (Figure 3A). Stearns Wharf bulk *Pseudo-nitzschia* abundances were significantly lower (average ~ 4.3 × 10^5^ cells L^−1^) compared to Goleta Pier (average ~ 5.0 × 10^5^ cells L^−1^) (Mann–Whitney U, *p* < 0.001), and both piers were lower on average than the offshore stations by at least a factor of three. Significant differences between piers and the offshore stations were only found at stations 4, 6, and 7 due to the high variability in offshore bulk *Pseudo-nitzschia* concentrations (Mann–Whitney U, *p* < 0.001). At Stearns Wharf and Goleta Pier, bulk *Pseudo-nitzschia* abundance and the wide class of *Pseudo-nitzschia* are significantly higher during the upwelling versus the non-upwelling time periods (*p* < 0.05). However, at the offshore stations, the high spatial and temporal variability in bulk *Pseudo-nitzschia* abundance resulted in no significant difference between the upwelling and non-upwelling periods (Kruskal–Wallis *p* > 0.05) (Figure 4A). It is important to note that while seasonal rains are often associated with increased nutrients from river discharge (data not shown, https://www.ncdc.noaa.gov/swdi), these rainfall and discharge events did not coincide with upwelling periods or with changes in bulk *Pseudo-nitzschia* abundance, dDA, or pDA concentrations (Kruskal–Wallis, *p* > 0.05), similar to previous studies in the basin [32,60] (Figure 4A,B). At the piers, there were significantly higher pDA concentrations during the upwelling versus the non-upwelling periods, which resulted in a significantly higher cDA during upwelling as well (Kruskal–Wallis test, *p* < 0.05) (Figure 4C). While the wide *Pseudo-nitzschia* were significantly correlated with pDA (Spearman’s rho, *p* < 0.01, linear regression, *p* < 0.05, adj. *r*^2^ = 0.203), narrow *Pseudo-nitzschia* were not (Spearman’s rho, *p* > 0.01, linear regression *p* > 0.05, adj. *r*^2^ = −0.005).

Particulate DA concentrations measured at Goleta Pier were significantly lower than those measured at either Stearns Wharf or the offshore stations (Mann–Whitney U, *p* < 0.001) (Figure 3C). The Stearns Wharf pDA, however, was not statistically different than the offshore stations, again due to the large variations in pDA concentrations measured. In the offshore stations, tDA surface water concentrations were similar between stations (Mann–Whitney U, *p* > 0.0013) and concentrations were relatively evenly distributed between the particulate (57.3 ± 34.8%) and dissolved (42.7 ± 34.8%) forms (average tDA = 7.8 × 10^3^ ± 2.2 × 10^4^ ng DA L^−1^, ranging from below detection (1.3 ng L^−1^) to 2.2 × 10^5^ ng DA L^−1^) (Figure 3, Table 1). Similar to bulk *Pseudo-nitzschia* cell abundance, surface tDA concentrations at the offshore stations were spatially and temporally variable with no seasonal or upwelling trends (Kruskal–Wallis, *p* > 0.05).

**Figure 5:**
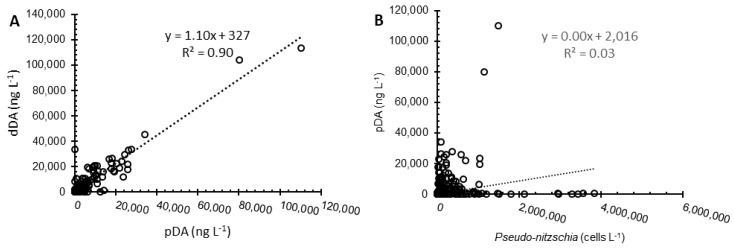
The dissolved domoic acid (dDA) versus particulate domoic acid (pDA) in ng L^−1^ for all water column data (1–7 at 0 m and Station 4, 0–150 m) collected from 2009–2013 (**A**). Particulate DA (pDA) in ng L^−1^ versus bulk *Pseudo-nitzschia* abundance (cells L^−1^) for all water column data (1–7 at 0 m and Station 4, 0–150 m) collected from 2009–2013 (**B**). All data are available in Table S3.

There is a significant correlation between pDA and *Pseudo-nitzschia* abundance in the offshore stations (Figure 5B, Spearman’s rho, *r* = 0.62, *p* < 0.01). However, the total *Pseudo-nitzschia* abundance is a poor predictor of pDA concentration (linear regression, adj. *r*^2^ = 0.03, *p* < 0.05) Figure 5B) across all piers (regardless of the *Pseudo-nitzschia* group) and transect stations. There was no temporal offset between surface bulk *Pseudo-nitzschia* and pDA concentrations either at a specific station or across all stations over the sampling period. In contrast, pDA and dDA concentrations across all transect stations and depths are significantly correlated (Spearman’s rho, *r* = 0.831, *p* < 0.01) and pDA is a strong predictor of dDA (linear regression, adj *r*^2^ = 0.90, *p* < 0.05 Figure 5A).

Concentrations of cDA ranged from <1.3 ng L^−1^ to 1400 pg cell^−1^ with higher cellular DA concentrations associated with the transect stations (average across all offshore stations = 37 ± 124 pg cell^−1^) relative to the Goleta Pier and Stearns Wharf (average across both piers = 18 ± 65 pg cell^−1^) (Mann–Whitney U, *p* < 0.05) (Figure 3E and Figure 4C). There is no significant difference in the mean cDA concentrations across the offshore stations or between the offshore stations and the piers (Mann–Whitney U, *p* < 0.001). There is no difference between the upwelling versus non-upwelling periods (Kruskal–Wallis, *p* > 0.05).

#### 2.1.2. Depth Distributions

**Figure 6:**
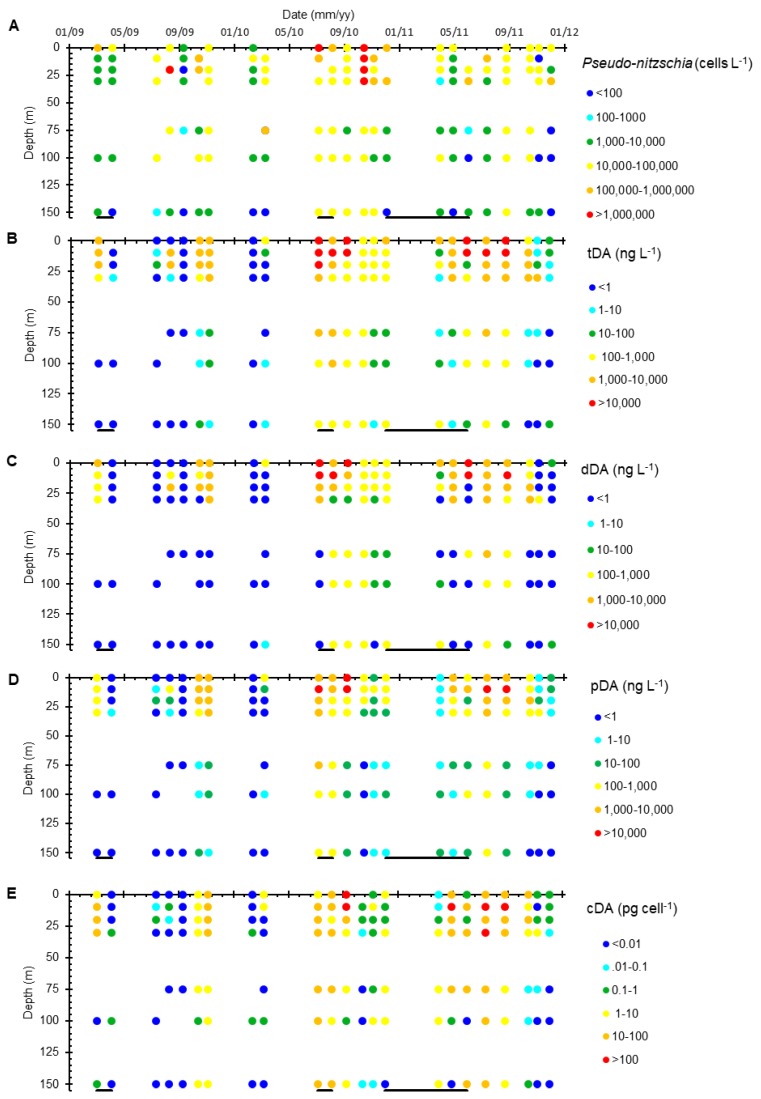
The bulk *Pseudo-nitzschia* abundance (cells L^−1^) (**A**), total domoic acid (tDA) concentrations (ng L^−1^) (**B**), particulate DA (pDA) concentrations (ng L^−1^) (**C**), dissolved DA (dDA) concentrations (ng L^−1^) (**D**), and cellular DA (cDA) concentrations (pg cell^−1^) (**E**) from 0–150 m at Station 4 from 2009–2011. Upwelling periods (12 °C isotherm shoals above 30 m) are identified by dark lines located underneath each panel. All data are available in Tables S1 and S3.

High bulk *Pseudo-nitzschia* abundances occurred in August 2009, July–December 2010, August 2012, and July 2013. At Station 4, *Pseudo-nitzschia* comprised >50% of the microphytoplankton (20–200 µm) community. Moderately high *Pseudo-nitzschia* abundances (>10^4^ cells L^−1^) were measured throughout the water column to depths as deep as 150 m multiple times during the sampling period (*n* = 6/23 at 150 m), particularly when the bulk *Pseudo-nitzschia* abundance at the surface was high (Figure 6A). During the upwelling periods, the bulk *Pseudo-nitzschia* abundance was generally greatest at the surface and decreased rapidly with depth (Figure 4D and Figure 6A). In contrast, during the non-upwelling periods, average *Pseudo-nitzschia* abundances were lower at the surface, such that the bulk abundances decreased more slowly with increasing depth (Figure 4D and Figure 6A). Bulk *Pseudo-nitzschia* abundances were significantly lower during the upwelling versus non-upwelling periods at 150 m (Kruskal–Wallis, *p* < 0.05).

At Station 4, the highest tDA concentrations (Figure 6B) typically occurred in the upper 25 m, although high tDA concentrations (>200 ng L^−1^) were observed as deep as 100 m several times (*n* = 5/21 at 100 m) throughout the time-series. Neither pDA or dDA concentrations were significantly different during the upwelling versus non-upwelling periods at any depth (Kruskal–Wallis *p* > 0.05) (Figure 4E). The total DA concentrations were generally higher during the upwelling periods (although not statistically significant due to high variability, Kruskal–Wallis *p* > 0.05) and rapidly decline with depth regardless of upwelling intensity (Figure 4D). Total DA concentrations also differ from *Pseudo-nitzschia* cell abundance in that peak tDA concentrations occurred both prior to (e.g., in August–September 2010) and following (e.g., September 2011) peaks in bulk *Pseudo-nitzschia* cell abundance. Furthermore, not all instances of high bulk *Pseudo-nitzschia* abundance resulted in an associated increase in tDA (*n* = 4, August 2009, February 2010, March 2010, November 2011 for high *Pseudo-nitzschia* cell abundance with low tDA concentrations) (Figure 6B).

Cellular DA concentrations at Station 4 ranged from below detection to as high as 320 pg cell^−1^ (average 18 ± 46 pg cell^−1^) and remained relatively constant down to 75 m, averaging 22 pg cell^−1^, before declining to less than 6 pg cell^−1^ below 100 m (Figure 4F and Figure 6). There was no significant difference between average cDA concentrations measured during upwelling versus non-upwelling periods above 150 m, (Kruskal–Wallis, *p* > 0.05, Figure 4F), but there is a significant difference at 150 m, where cDA concentrations measured during non-upwelling (9.4 pg cell^−1^) were almost double those measured during upwelling (5.1 pg cell^−1^) (Kruskal–Wallis *p* < 0.05).

### 2.2. Water Column Inventories and Flux Comparison

**Figure 7:**
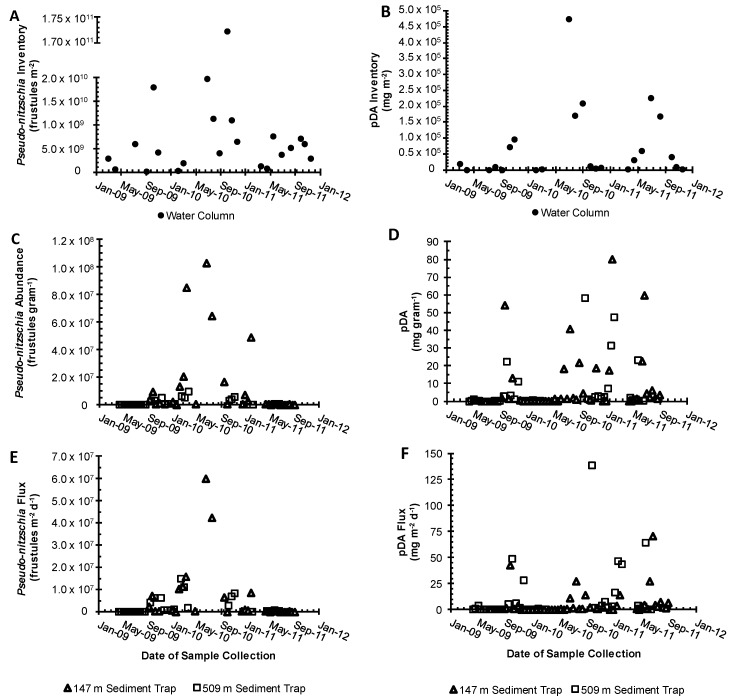
The bulk *Pseudo-nitzschia* * (cells m^−2^) (**A**) and particulate domoic acid (pDA) (mg m^−2^) inventories (**B**) integrated over the upper 150 m of the water column (black circles) from 2009–2011. Inventories were determined by multiplying *Pseudo-nitzschia* abundance or the pDA concentration by the depth interval between the midpoint of the overlying sampling depth and the midpoint of the sampling depth below the targeted depth and adding these products together down to 150 m. Bulk *Pseudo-nitzschia* abundance (frustules g sediment^−1^) (**C**), pDA concentration (mg g sediment^−1^) (**D**), bulk *Pseudo-nitzschia* flux (frustules m^−2^ day^−1^) (**E**), and pDA flux (mg m^−2^ d^−1^) (**F**) into the 147 m (triangles) and 509 m (squares) sediment traps from 2009–2011. * Note the break in the *y*-axis of panel A. All sediment trap data are available in Table S4.

Temporal differences in sampling (one day versus two weeks) make comparisons between water column sampling and sediment trap concentrations and fluxes difficult. Nonetheless, comparisons were made in an attempt to identify specific processes influencing both data sets. Inventories of bulk *Pseudo-nitzschia* cell abundance and pDA concentrations in the water column (0–150 m) were calculated for each cruise and compared to the corresponding sediment trap collection period (Figure 7A). There were no significant correlations between the bulk *Pseudo-nitzschia* and pDA inventories and the bulk *Pseudo-nitzschia* and pDA concentrations and fluxes measured in either of the sediment traps (Spearman’s rho, *p* > 0.01). Less than 5% of water column inventories were captured per day (the inventory divided by daily flux) in either trap throughout the sampling period (Figure 7).

### 2.3. Sediment Traps

During the study period, there are two significant periods of missing data from the 509 m trap due to clogging (April–September 2010 and July–October 2011). This is not an uncommon occurrence and is often associated with high flux events that interfere with the trap cup rotation [32]. The average concentration of *Pseudo-nitzschia* in the 147 m trap was 1.21 × 10^7^ ± 2.62 × 10^7^ frustules g sed.^−1^ and ranged from 1.17 × 10^3^ to 1.03 × 10^8^ frustules g sed.^−1^ (Figure 7C). These concentrations are not significantly different than those measured in the 509 m trap due to a high variability (Kruskal–Wallis *p* > 0.05). Bulk *Pseudo-nitzschia* concentrations averaged 2.04 × 10^6^ ± 2.64 × 10^6^ frustules gram sed.^−1^ and ranged from 8.22 × 10^3^ to 9.54 × 10^6^ frustules g sed.^−1^. Particulate DA concentrations in each trap were also not significantly different (Kruskal–Wallis *p* > 0.05) and ranged from below detection to 79.9 µg pDA g of sed.^−1^ and averaged 8.4 ± 17.3 and 8.0 ± 15.0 µg pDA g of sed. ^−1^ in the 147 and 509 m sediment traps, respectively (Figure 7D, Table 1). It is important to note that DA degradation occurs in the sediment traps throughout deployment, such that sediment trap pDA concentrations may be underestimated by as much as 50% [32]. There was no relationship between sediment trap bulk *Pseudo-nitzschia* abundance and DA concentration.

Sediment trap fluxes varied considerably throughout the times-series, with the flux of bulk *Pseudo-nitzschia* ranging from 523 to 6.00 × 10^7^ frustules m^−2^ d^−1^ and averaging 5.37 × 10^6^ ± 1.31 × 10^7^ frustules m^−2^ d^−1^ in the 147 m trap. Particulate DA in the 147 m trap ranged from BD to 70.8 µg DA m^−2^ d^−1^ and averaged 5.6 ± 13.0 µg DA m^−2^ d^−1^. The flux of the bulk *Pseudo-nitzschia* into the 509 m trap was significantly lower than that measured at shallower depths, ranging from 7.59 × 10^3^ to 1.48 × 10^7^ frustules m^−2^ d^−1^ and averaging 3.07 × 10^6^ ± 4.13 × 10^6^ frustules m^−2^ d^−1^ (Kruskal–Wallis *p* < 0.05) (Figure 7C). In contrast, pDA fluxes showed the opposite trend, with significantly higher pDA fluxes at depth, averaging 5.6 versus 15.2 µg pDA m^−2^ d^−1^ (Kruskal–Wallis *p* < 0.05). Given the similarities in pDA concentrations between the two depths, differences in flux were almost entirely driven by the total mass flux (0.7 versus 1.7 g m^−2^ d^−1^ at 147 and 509 m, respectively).

Although the data were limited, both traps show periods of high pDA and *Pseudo-nitzschia* fluxes in mid-winter and summer of each year (Figure 7C, D). There were significant differences between bulk *Pseudo-nitzschia* concentrations and fluxes during upwelling (2.29 × 10^7^ cell g sed.^−1^, 9.98 × 10^6^ frustules m^−2^ d^−1^) versus the non-upwelling periods (2.00 × 10^6^ cells g sed.^−1^, 1.06 × 10^6^ frustules m^−2^ d^−1^) in the 147 m trap, and in *Pseudo-nitzschia* flux (2.66 × 10^6^ frustules m^−2^ d^−1^ versus 3.48 × 10^6^ frustules m^−2^ d^−1^, upwelling and non-upwelling, respectively) in the 509 m trap (Kruskal–Wallis *p* < 0.05) when compared directly to surface waters. When the trap data lagged two weeks behind the start of the upwelling period, bulk *Pseudo-nitzschia* concentrations in the 509 m trap were significantly higher during upwelling as well (Kruskal–Wallis, *p* < 0.05). Note that there was no change in significance in the bulk *Pseudo-nitzschia* flux data for either trap.

Particulate DA concentrations and fluxes were characterized by different trends. In both the 147 m and 509 m traps, pDA concentrations were not significantly different during upwelling versus non-upwelling with no time lag, or with a 2-week delay in the trap compared to the surface (Kruskal–Wallis, *p* > 0.05). In the 147 m trap during non-upwelling, the average pDA concentration was 11.0 µg pDA per g sed. and the average pDA flux was 10.7 µg DA m^−2^ d^−1^. During upwelling, the average pDA concentration was similar, 10.7 µg per g sed., and the average pDA flux was more than two times lower, 4.2 µg m^−2^ d^−1^. Differences were not significant due to the high variability in the data. In the 509 m trap during non-upwelling, the average pDA concentration was 3.8 µg pDA per g sed. and the average pDA flux was 8.1 µg DA m^−2^ d^−1^. During upwelling, the average pDA concentration was similar, 9.3 µg per g sed., and the average pDA flux was more than two times lower, 15.1 µg m^−2^ d^−1^. Again, differences were not significant due to the high variability in the data.

## 3. Discussion

### 3.1. Water Column

Toxic *Pseudo-nitzschia* blooms occur worldwide and are commonly associated with eastern boundary currents, such as along the West Coast of the United States where the upwelling of nutrient-rich waters is supplemented by nutrients from riverine runoff and upwelling induced mesoscale circulation patterns that promote diatom growth [1,15,17,61,62,63]. Within the SBB, toxic *Pseudo-nitzschia* blooms have become a regular occurrence since they were first observed in 1998 when a widespread bloom resulted in the mass mortality of more than 400 sea lions [3,5]. Since then, efforts to understand the mechanisms that foster toxic *Pseudo-nitzschia* blooms have proliferated [1,12], along with attempts to develop predictive models of when and where *Pseudo-nitzschia* blooms will occur [15,16,64].

Numerous studies have linked bulk *Pseudo-nitzschia* abundance and growth rates along the western United States to increased nutrients supplied by seasonal upwelling conditions [3,7,48,65]. In the SBB, the dominant species in the basin, *Pseudo-nitzschia australis* [15,32,50], is associated with the warming of cold, high salinity upwelled waters, and peaks in abundance late within the primary diatom bloom season [7,16]. These increases in *Pseudo-nitzschia australis* concentrations are further associated with a decline in nutrient availability in source waters [13,59] and warming ocean temperatures on longer time scales [9,46].

In this study, we observed wide temporal and spatial variations in bulk *Pseudo-nitzschia* abundance and spatial distributions that did not correspond with the upwelling conditions. Instead, bulk *Pseudo-nitzschia* were almost always present in the SBB with bulk *Pseudo-nitzschia* generally occurring in the upper 20 m of the water column in a range of cellular abundances similar to those found in other studies in this area [7,15]. While upwelling data are limited relative to our non-upwelling sampling, results suggest that the lack of seasonal differences in bulk *Pseudo-nitzschia* abundance are more likely due to the climate-induced warming of waters [9,46,50] or due to biogeochemical changes in the ambient nutrient regime (i.e., low Si:N and N:P ratios) that allow bulk *Pseudo-nitzschia* to maintain a robust population year-round [59].

Spatially, surface *Pseudo-nitzschia* abundances observed offshore rarely matched those measured in the pier-based sampling stations (Goleta and Stearns Wharf Piers) or even in the adjacent transect stations, often differing by more than an order of magnitude. Some of these differences may be due to differences in sample timing at offshore versus pier stations and the relatively fewer samples offshore. However, these results are consistent with results from a skill assessment of the California Harmful Algae Risk Mapping (C-HARM) System that showed that weekly samples from Stearns Wharf were decoupled from 3-km pixel predictions of bulk *Pseudo-nitzschia* blooms and DA concentrations [64]. This model also supports our observations that environmental conditions are suitable for year-round *Pseudo-nitzschia* growth in offshore waters.

At least some of this nearshore–offshore difference may be attributed to the physical circulation patterns related to mesoscale eddies, cyclonic currents, and the progression of upwelling within the central SBB [15,55], as well as potential diversity in *Pseudo-nitzschia* spp. For example, Bialonski et al. [66] found that the SBB circulation dynamics play a role in transporting phytoplankton from one area of the basin to another. While some of the sampling sites were considered sources of phytoplankton seed populations, the source areas changed seasonally and annually, suggesting that allochthonous sources may also influence *Pseudo-nitzschia* abundance. Regardless, during our study, offshore waters that have been previously under-sampled revealed perennial *Pseudo-nitzschia* populations.

The single depth profiles in the center of the SBB also reveal that *Pseudo-nitzschia* inhabits the entire mixed layer, with significant cell abundances (>50,000 cells L^−1^) occurring below the surface at 10–30 m in depth. The presence of these subsurface *Pseudo-nitzschia* supports the hypothesis put forth by Seegers et al. [67] that subsurface *Pseudo-nitzschia* could “seed” surface blooms following upwelling events. These “hidden” or “cryptic” blooms are prevalent worldwide, with thin layers of toxic *Pseudo-nitzschia* measured in nearby Monterey Bay at depths of 10–15 m [29,68]. The presence of significant deep *Pseudo-nitzschia* cell abundances (> 10^4^ cells L^−1^), at times reaching 150 m (our deepest water column sampling point), are almost always associated with a high *Pseudo-nitzschia* abundance in overlying surface waters (*n* = 6). We hypothesize that these deep *Pseudo-nitzschia* cells are likely the result of aggregation and flocculation [69], consistent with Timmerman et al. [29], who further demonstrated that thin layers were dominated by diatom flocs, as opposed to single cells.

The toxicity of *Pseudo-nitzschia* within the SBB is more complex (Figure 3 and Figure 6). Shore-based tDA concentrations were consistently two to three orders of magnitude lower than those measured offshore (e.g., November 2009 and July 2012; Figure 3B). While these average tDA concentrations were generally higher than those previously measured in this region, they were well within the range of the reported tDA concentrations [7,9,16]. In the central SBB (Station 4), the highest tDA concentrations occurred before (e.g., August to September 2010), during (June and July 2011), and after (October to November 2009) the peak in bulk *Pseudo-nitzschia* cell abundance, with no relationship to seasonal upwelling. Furthermore, significant water column tDA concentrations (>200 ng L^−1^) were measured as deep as 100 m (Figure 6). These findings argue that bulk *Pseudo-nitzschia* cell abundance is a poor predictor of toxicity and that measuring DA concentrations independently of *Pseudo-nitzschia* abundance is needed, as DA may be present in the water column in the absence of abundant *Pseudo-nitzschia* at the surface, i.e., summer 2011.

Cellular DA concentrations varied considerably, with an average of 18.3 ± 43.6 pg cell^−1^ at Station 4 over all depths, and an average of 37 ± 124 pg cell^−1^ at surface Stations 1–7. Cellular DA concentrations were generally within the range of the published values (0–117 pg cell^−1^), although among the higher end of those previously measured, the maximum cDA found here (1400 pg cell^−1^) is higher than any previously published values [1,9,16,17,70]. However, unlike tDA or bulk *Pseudo-nitzschia* abundance, cDA concentrations were relatively constant with increasing depth down to 75 m (Figure 6). Consistent with previous work, bulk *Pseudo-nitzschia* toxicity in the SBB over our short sampling period appears to depend on a variety of factors other than bulk *Pseudo-nitzschia* cell abundance [12]. While the exact mechanisms that promote *Pseudo-nitzschia* toxicity are complex [12], the majority of research along the West Coast of the United States argues that increasing toxicity occurs in response to increasing physiological stress and is often associated with the stationary phase of cell growth [1]. In laboratory cultures, Schnetzer et al. [27] showed that the cellular toxicity of *P. australis* increased by an order of magnitude as the bloom progressed from the exponential growth phase to the senescence and marine snow formation (See Section 3.2). Environmental factors such as temperature likely facilitate cell toxicity as well, although it can be difficult to separate temperature from the nutrients and growth phase. For example, Anderson et al. [51] found that diatom dominant assemblages and increased bulk *Pseudo-nitzschia* abundances occur at the end of the upwelling season in the Santa Barbara Channel when nutrients were still plentiful, but waters were warming. In follow-up studies, Anderson et al. [16] found that models driven by lower Si:N ratios, which occur towards the end of a diatom bloom, were the best predictor of DA toxicity in the SBB. In their study of the largest DA-producing bloom measured along the western United States, McCabe et al. [9] found that the *Pseudo-nitzschia* bloom was initiated by nutrients from upwelled waters and then sustained by warmer temperatures once upwelling ceased. Similar results have been found internationally. Off the coast of Namibia, Louw et al. [45] found the highest bulk *Pseudo-nitzschia* abundances to occur at the more moderate temperatures following upwelling events and Dursun et al. [71] found that DA concentrations in a Turkish estuary increased with increasing temperature.

Species and strain identification of *Pseudo-nitzschia* spp. were beyond the scope of this work, but the variation in pDA production by species and strains likely plays an important role in the decoupling observed between pDA concentrations and bulk *Pseudo-nitzschia* biomass in the SBB during our study period [1,72,73]. While highly toxigenic strains of *P. australis* and *P. multiseries* have been identified in SBB blooms [1,32], so have less toxigenic species, e.g., *P. fraudulenta* [1]. Using samples from the SBB, Seubert et al. [52] found that differentiating between these two size classes, which is possible via light microscopy, provides a rough approximation of the presence of highly toxic versus less toxic or non-toxic species when more precise methods of species identification are not available. At the pier stations, where size classes were used to differentiate between rarely toxic and usually toxic cells, wide *Pseudo-nitzschia* (often toxic, e.g., *P. australis*) was significantly correlated with pDA (Spearman’s rho, *p* < 0.01) while narrow *Pseudo-nitzschia* (rarely toxic, e.g., *P. delicatissima*) was not (Spearman’s rho, *p* > 0.01). In the nearby San Pedro Channel, Smith et al. [74] found similar results. While bulk *Pseudo-nitzschia* abundance increased after medium strength upwelling intervals, pDA concentrations did not. Rather, Smith et al. [74] found that DA concentrations were strongly influenced by *Pseudo-nitzschia* speciation, with higher pDA concentrations occurring with more toxigenic species (based on ribosomal analyses) towards the end of an upwelling event when silicic acid concentrations had declined (along with other nutrients). In the SBB, the relative dominance of the wide and narrow *Pseudo-nitzschia* varied temporally. However, only the abundance of the wide size class was significantly higher during upwelling. These results are therefore consistent with previous work in the SBB and elsewhere linking the appearance of potentially toxic *Pseudo-nitzschia* with upwelling to post-upwelling conditions, at least in the nearshore [52].

Particulate DA concentrations and toxic *Pseudo-nitzschia* have been the focus of most extant studies due to potential impacts on human health due to the bioaccumulation of toxic particles by shellfish populations. However, our results confirm that dDA is a significant component of the tDA pool. Total DA concentrations were almost evenly distributed between the dissolved and particulate phases (Figure 5A). Previous investigations of dDA and pDA concentrations suggest that their distribution may be species-dependent. In a study of a *P. cuspidata* bloom off the coast of Washington state, Trainer et al. [63] found that partitioning between pDA and dDA was highly variable. In contrast, Baugh et al. [70] found that more toxic *Pseudo-nitzschia* spp. produced significantly higher pDA concentrations relative to dDA. In field samples containing ten times a lower amount of *P. australis* (highly toxigenic) than *P. delicatisssima* (barely toxigenic), pDA concentrations were half that of dDA concentrations [70]. In field samples with approximately equal concentrations of *P. australis* and *P. delicatissima*, pDA and dDA concentrations were similar [70]. While variations in the pDA:dDA ratio did occur in our study (Figure 5A) and may be due to changes in the specific mixture of *Pseudo-nitzschia* species present or other factors affecting bloom toxicity, the strong linear regression between dDA and pDA indicates that variations are relatively small, regardless of when the samples were collected or the species present. Our results of a near equal distribution of pDA and dDA within the SBB during our field campaign, therefore, suggests that *Pseudo-nitzschia* was likely comprised of a more even mixture of toxic and less toxic *Pseudo-nitzschia* spp., consistent with the pier results.

The existence of high amounts of dDA throughout the upper 75 m of the water column that persists for several months is an intriguing finding (Figure 6C). Once produced, DA is water-soluble and has little to no particle reactivity at the particle loads typical of the marine water column [75]. Dissolved DA may photochemically degrade with degradation rates declining rapidly with depth [28,76]. The rate of degradation, however, depends on the depth of light penetration, temperature, and potentially dissolved organic matter and iron concentrations [28,77], such that degradation rates exponentially decrease with increasing water depth and are essentially negligible by 5 m due to light attenuation [28]. Bacteria also likely degrade DA depending on the bacterial assemblages present. However, information is limited as studies either focus on bacteria associated with specific species of *Pseudo-nitzschia*, e.g., *P. multiseries* [25,31,78], or with higher trophic levels, i.e., bacteria from blue mussels, sea scallops, and anchovies [78,79]. Bacterial degradation rates are likely underestimated, as current studies of photochemical versus bacterial degradation do not completely eliminate the influence of the other pathway [31]. Our results suggest that while dDA may undergo degradation, significant dDA concentrations remain present throughout the upper water column (Figure 5A and Figure 6C). These results also suggest that dDA and pDA concentrations have comparable residence times, such that dDA loss via degradation and pDA loss (via sinking) from the water column must be similar.

The ecosystem impact of dDA remains ambiguous, specifically the potential effect on feeding behavior, bioaccumulation, or the health of higher-trophic-level animals. Current research has shown dDA exposure can cause developmental defects in zebrafish and scallops [38,40], and lower krill grazing rates [39]. Van Meerssche and Pinkney [41] found that dDA, in concert with salinity, inhibits the growth of some phytoplankton groups in an allelopathic manner. Regardless of the mechanism and potential direct impact on biota, dDA concentrations may provide an indication of the *Pseudo-nitzschia* spp. present in the SBB water column and potential toxicity, i.e. the pDA available for bioaccumulation. These results are particularly valuable given the recent developments of *in situ* techniques for the rapid monitoring of dDA concentrations [80,81].

### 3.2. Surface to Depth Transport

The absolute magnitudes of *Pseudo-nitzschia* and pDA inventories in the water column, as well as the concentrations and fluxes measured in the sediment traps, varied significantly, with less than 5% of water column inventories captured on a daily basis in either sediment trap throughout the sampling period. This is not surprising as water column particles span a range of sizes and densities that may or may not sink prior to remineralization. These results, however, are lower than that of Krause et al. [54] who found that, on average, 10% of the biogenic silica (which includes *Pseudo-nitzschia* frustules) measured in the upper 75 m within the SBB is exported to the deep sediment trap on a daily basis. Lower biogenic silica export efficiencies are likely related to plankton composition. While *Pseudo-nitzschia* may dominate the microphytoplankton communities during a bloom event, they are not the only diatom or siliceous organisms present in the system. For example, *Chaetoceros* spp., *Rhizosolenia-*related *spp.*, and silicoflagellates are also common during SBB blooms [50,82,83]. Furthermore, *Pseudo-nitzschia* spp. are generally lightly silicified and therefore subject to more rapid and extensive dissolution upon cell senescence and death [62,84]. Remineralization of *Pseudo-nitzschia* is supported by the average differences in *Pseudo-nitzschia* frustule abundance and fluxes between the two sediment traps, with deep traps containing *Pseudo-nitzschia* frustule concentrations more than 5 times lower and fluxes that are half that observed in the shallow trap.

Previous work in the SBB has demonstrated strong seasonal differences in the flux of nutrients, carbon, and opal to the deepest trap, with significantly higher fluxes occurring during upwelling (*p* < 0.001, [85]), consistent with peak plankton biomass. Krause et al. [54] found that peaks in opal fluxes in the deep sediment trap lagged peaks in upper water column opal (diatom) inventories by two weeks to two months. Sekula-Wood et al. [30], however, found no such lag between bulk surface water *Pseudo-nitzschia* abundance and pDA concentrations and fluxes measured as deep at 800 m in the SBB and San Pedro Basin, and argued for rapid transport, with particle sinking rates in excess of 100 m d^−1^. Here, there was a significant difference between the sediment trap upwelling and non-upwelling bulk *Pseudo-nitzschia* concentrations and flux when either no delay or a two-week delay was imposed on the deep sediment trap data. Hence, our results are consistent with Krause et al. [54] and Sekula-Wood et al. [30] in that bulk *Pseudo-nitzschia* fluxes were rapidly transported to the depth, at rates >50 m d^−1^.

The difference in water column export efficiency between biogenic silica, *Pseudo-nitzschia*, and pDA in the sediment traps likely occurs for several reasons including spatial and temporal heterogeneity, variations in remineralization patterns, and issues associated with water column and sediment trap collection methods. First and foremost, spatial sampling of the SBB as a whole suggests that single day and point depth profiles do not capture the full temporal and spatial variability of pDA and *Pseudo-nitzschia* cell abundance occurring in the SBB. For example, *Pseudo-nitzschia* blooms form aggregate layers below the surface along isopycnals that persist on the order of days to weeks [29,86]. These thin layers are easily missed by conventional water column sampling.

It is also possible that subsurface *Pseudo-nitzschia* are horizontally advected to other areas of the basin before reaching the sediment traps and underlying sediments, similar to how surface blooms can be transported horizontally (see Section 3.1). The SBB is host to an array of complex circulation patterns that have the potential to transport blooms from one area of the basin to another, including mesoscale eddies and wind-induced cross-shelf transport [15,55,66]. Horizontal advection of blooms could, therefore, decouple upper water column *Pseudo-nitzschia* and pDA from those measured in the sediment traps located directly below.

Differences in water column inventories and fluxes into the shallow and deep sediment traps and between traps may also be due to variations in trap collection area and efficiency. Shallow traps suffer from hydrodynamic effects associated with the advective shear of water flow over the trap surface [87]. While the SBB traps use baffles to reduce this sheer, both the under- and over-collection of trap material may occur. Due to the depth difference between the surface and deep traps, the deep trap has a collection area two to four times larger depending on where in the upper water column particles originate. Given the spatial heterogeneity observed across the SBB, it is quite possible that the surface and bottom traps collect different material. This is most evident in the pDA. While pDA concentrations are similar between trap depths, pDA fluxes into the deeper 509 m trap, (15.2 µg DA m^−2^ d^−1^) were almost three times higher than that measured at 147 m and were almost entirely driven by differences in the total mass flux (0.7 versus 1.7 g m^−2^ d^−1^ at 147 and 509 m, respectively). Krause et al. [54] argued that a significant fraction of the biogenic silica flux measured in the deepest SBB trap originated from outside of the SBB and was likely advected into the SBB via strong and seasonally changing coastal currents. Unfortunately, our limited data set does not allow us to differentiate external versus locally sourced sinking particles.

Bulk *Pseudo-nitzschia* fluxes decrease by almost a factor of two at the depth. This suggests that either bulk *Pseudo-nitzschia* are much more rapidly remineralized than particle-associated DA or that toxic *Pseudo-nitzschia* are much more efficiently exported, perhaps through repackaging in rapidly sinking zooplankton fecal pellets [88]. In addition, *Pseudo-nitzschia* also produce transparent exopolymers (TEP) during physiological stress and bloom decline [35,89,90]. TEP are a critical component of diatom flocculation and the formation of aggregates, a key precursor to sinking (e.g., [91]). A recent laboratory study by Schnetzer et al. [27] found that DA production within *P. australis* occurred rapidly during marine snow formation and in response to nitrogen stress, with pDA loss rates of less than 2% d^−1^. Thus, we argue that toxic *Pseudo-nitzschia* may also be more effectively and rapidly transported to the depth within the SBB due to the similar mechanisms that promote DA and marine snow formation.

Although <5% of the water column DA inventory sinks to the depth on a daily basis, sinking particle concentrations and fluxes are still cause for environmental concern. Particulate DA concentrations in the 147 and 509 m sediment traps averaged 8.4 and 8.0 µg pDA grams of sed.^−1^, respectively, with six events exceeding 20 µg per g sed. between January 2009 and June 2012. Again, this is considered a minimum estimate as DA is known to degrade in sediment trap cups [32]. Thus, sinking particles laden with DA are a likely source of the toxin to pelagic and benthic food webs even when no surface *Pseudo-nitzschia* blooms have been observed [6,9,36,37]. The most dramatic example of such a benthic contamination was observed in the closure of the Dungeness and rock crab fisheries along the U.S. West Coast during the 2015–2016 season due to high DA levels in crab tissues. At least 49 million dollars in revenue was lost in California alone [92]. Mitigating losses due to such closures is a key goal of harmful algal bloom analysts and policymakers.

## 4. Conclusions

*Pseudo-nitzschia* blooms occur worldwide, and as blooms increase in frequency and new species are described, it has become increasingly important to have effective tools for monitoring and modeling toxic blooms and their impact on ecosystems and economies. Bulk *Pseudo-nitzschia* blooms were not significantly correlated with the upwelling versus non-upwelling conditions. While tDA concentrations tended to be higher during upwelling conditions and there was a significant correlation between tDA and *Pseudo-nitzschia* abundance in offshore waters, the total *Pseudo-nitzschia* abundance was a poor predictor of pDA concentration across all stations. This is different from previous work that has shown that toxic *Pseudo-nitzschia* blooms typically occur immediately following coastal upwelling events throughout the world, in Southern Africa, Western Europe, South America, and the United States [1,7,44,45,46,47]. In this study, bulk *Pseudo-nitzschia* concentrations are spatially and temporally heterogeneous and are always present in the SBB. These results, combined with the *Pseudo-nitzschia* size classifications measured at the pier stations indicates that a variety of bulk *Pseudo-nitzschia* spp. exist in the SBB, and that variability at the species and strain level likely drives toxicity in response to a suite of environmental conditions, such as upwelling, that are complicated by the bloom stage.

This study contributes to the growing body of evidence that dDA is a significant component of water column DA concentrations. *Pseudo-nitzschia* blooms and pDA and dDA concentrations were often found throughout the upper water column, with significant water column DA concentrations measured at depths as deep as 150 m. The combination of relatively high pDA and high concentrations of dDA supports the argument that DA in all phases should be considered in studies of DA allelopathic and ecosystem effects. Given the predictable partitioning of DA between particulate and dissolved phases, dDA may further serve as an indicator of toxigenic *Pseudo-nitzschia* spp. presence when no other measurements are available. Despite decoupled water column inventory and sediment trap measurements, the flux of DA to depth is relatively rapid, with minimal degradation as particles sink through the water column. We argue that this flux is likely due to the formation of toxic marine snow that occurs with bloom senescence and results in significant DA export to the seafloor. Our work adds to the growing body of literature on DA toxicity in marine ecosystems and highlights the necessity of measuring both dissolved and particulate DA forms, *Pseudo-nitzschia* spp., and water column measurements below the surface. Although only a small fraction of DA produced in the water column reaches the seafloor, our results confirm that concentrations remain significantly high that monitoring benthic organisms in this region should occur regularly. Sampling throughout the year in the SBB should be continued to confirm results over longer timescales in response to changing environmental factors.

## 5. Materials and Methods

### 5.1. Pier Stations

Pier station data were collected at Stearns Wharf (34°24.48′ N, 119°41.10′ W) and Goleta Pier (32°52.02′ N, 117°15.42′ W) in Santa Barbara, CA through the Southern California Coastal Ocean Observing System (SCCOOS, Figure 1) HAB monitoring program. The pier data used in this study were collected from January 2009 to December 2013. Samples were collected weekly and analyzed for pDA, *Pseudo-nitzschia* abundance, and a suite of other water quality parameters in accordance with SCCOOS and Harmful Algal Bloom Monitoring and Alert Program (HABMAP) monitoring protocols [52]. Water samples for pDA were filtered through GF/F filters and pDA was measured using the ELISA bioassay [52]. Dissolved DA was not measured, therefore tDA data are not available. Cellular DA concentrations were determined by dividing the measured pDA by the total *Pseudo-nitzschia* abundance. *Pseudo-nitzschia* were divided into two size classes and designated as either the *P. delicatissima* type (frustule widths <3 µm) or the *P. seriata* type (frustule width >3 µm) [52]. The reason for the size class distinction is to provide a rough estimate of toxigenic species abundance in lieu of routinely using scanning electron microscopy to definitively identify *Pseudo-nitzschia* at the species level. *P. delicatissima*, or the narrow size class, is rarely associated with toxic blooms, while *P. seriata*, or the wide size class, may contain highly toxigenic species, such as *P. multiseries* and *P. australis* [52]. As no scanning electron microscopy was conducted during this study, we use narrow and wide to describe the *Pseudo-nitzschia* size class data collected at the piers.

### 5.2. Offshore Stations

Water samples were collected monthly at seven stations located along a transect through the middle of the SBB as part of the University of California Santa Barbara Plumes and Blooms project from March 2009–June 2013 (Figure 1). Only surface samples were collected at stations 1–3 and 5–7 using Niskin bottles attached to a standard rosette. At Station 4, located in the center of the SBB (34°15′ N, 119°54′ W), samples were typically collected at seven standard depths (0, 10, 20, 30, 75, 100, and 150 m) as well as at the deep chlorophyll maximum. Samples from Niskin bottles were collected for dDA and pDA concentrations, and bulk *Pseudo-nitzschia* cell abundances. Conductivity, temperature, and depth (CTD) profiles and photosynthetically active radiation (PAR) were also measured at all stations as part of the Plumes and Blooms core measurements using the methods described in Anderson et al. [51]. Bulk *Pseudo-nitzschia* samples were preserved in borate buffered formalin (Fisher Scientific, Hampton, NH, USA) with a final concentration of 2% and cell abundances were counted using the Utermöhl method [30,93]. Aliquots (10 mL) were settled and cells over 5 µm were counted at a 400X magnification with a minimum cell count of 100 cells per sample. The effective detection limit of this method is 200 cells L^−1^ [94]. Unlike the pier stations, there was no distinction made between narrow or wide *Pseudo-nitzschia* frustules in the offshore station samples. Approximately 500 mL seawater samples were filtered through a Whatman 25 mm GF/F and the filters were immediately frozen and stored at −80 °C. Whole water samples were gently filtered using Whatman 0.45 µm GF/F syringe filters into acid cleaned scintillation vials and stored refrigerated prior to the measurement of dDA. Particulate and dissolved DA concentrations were measured for each water sample using the method described in Sekula-Wood et al. [30,32]. Briefly, samples were analyzed using tandem Liquid Chromatography-Mass Spectrometry (LC-MS/MS) with an Agilent 1100 high-performance liquid chromatography coupled to a Micromass-Quattro mass spectrometer equipped with an electrospray ion-spray interface. The mobile phase was trace metal grade 0.1% formic acid in deionized water and 0.1% formic acid in acetonitrile from Fisher Scientific with a detection limit of 1.3 ng DA mL^−1^. Total DA (tDA) concentrations are the sum of pDA and dDA concentrations. Cellular DA (cDA) is calculated as the amount of pDA per total *Pseudo-nitzschia* cell abundance in a known volume. To better link water column observations to the concentration and fluxes of material captured in the moored sediment traps, *Pseudo-nitzschia* cell abundance and pDA concentrations from the upper 150 m of the water column at Station 4 were depth-integrated by the trapezoidal rule using the midpoint between sample depths and the measured concentrations at those sample depths. In other words, the mid-depth between specific sampling points was used to define a depth range. The average concentration at this mid-depth was then multiplied by the depth range to obtain an integrated concentration. These integrated “boxes” were then summed over a specific depth interval of interest to determine an inventory of DA (mg DA m^−2^) or *Pseudo-nitzschia* cell abundance (cells m^−2^) [63].

### 5.3. Sediment Traps

Sinking particulate samples were obtained from two moored Mark IV sediment traps deployed at 147 ± 2 and 509 ± 23 m located near the center of the SBB (34°14′ N, 120°2′ W) in a total water depth of ~590 m (Figure 1). In the shallow 147 m trap, samples for this study were collected from October 2009–October 2011. Deep trap (509 m) sample collection began in August 1993 with data collected from 1993 to 2008 reported in Sekula-Wood et al. [32], and data from 2009–2012 reported here. Gaps in sediment trap data are due mainly to trap clogging, failure to retrieve the sediment trap, and to a much lesser extent, the insufficient sample size because of very low mass flux. Trap deployments lasted approximately six months with each trap cup (*n* = 13) collecting sediment continuously for ~two-week periods. The sample cups were deployed filled with filtered seawater containing a solution of 10% sodium azide and 1% sodium borate (Fisher Scientific) for sample preservation [32]. Sediment trap samples were analyzed for *Pseudo-nitzschia* cell abundance using the Utermöhl method described above [30,93]. Particulate DA within freeze-dried and ground sediment was extracted using 1.3 mL of 50% methanol (Fisher Scientific) and analyzed as detailed in Sekula-Wood et al. [30,32]. Sediment trap supernatant solutions were filtered and measured directly using the same process as the water column dDA samples described above. Particulate DA (pDA) is the sum of the supernatant dDA and particle DA measured within the sediment trap normalized for supernatant volume and sediment trap mass. Sediment trap fluxes of *Pseudo-nitzschia* and pDA were determined by multiplying their concentration by the total grams of sediment captured during the ~two-week collection period into the 0.5 m^2^ opening of each sediment trap and dividing that by the number of days in the collection period.

### 5.4. Statistics

All statistical analyses were done using IBM SPSS Statistics Version 24. None of the dependent variables were normally distributed and data transformations (e.g., Log(x + 1), 1/x, √x, etc.) resulted in non-normal distributions. Because variances were not homogeneous and data were not normally distributed, significance testing required the use of non-parametric tests. For comparisons between non-upwelling and upwelling conditions, the Kruskal–Wallis test was used to determine significant differences, and the level of significance was set at 0.05. Correlations were determined using Spearman’s rho testing, and the level of significance was conservatively set at 0.01. Mann–Whitney U testing was conducted to determine the differences between surface stations with a Bonferroni correction setting the significance level at <0.0014. Linear regressions were performed to determine the mathematical relationships between pDA, dDA, tDA, and *Pseudo-nitzschia* abundance. DA samples below the detection limit were represented by 0′s, except in 3 cases where a non-zero number below the detection limit was used. In some cases, *Pseudo-nitzschia* cell counts were below the effective detection limit of 200 cells L^−1^ set by Hallegraeff et al. [94]. When this occurred (*n* = 11), the number of cells actually counted was used for statistical calculations.

## Figures and Tables

**Table 1:** The number of measurements (N), minimum (Min.), maximum (Max.), median, mean, and standard deviation (Std. Dev) of all data (2009–2013) collected at the surface stations (1–7 at 0 m), Station 4 depth profiles (0–150 m), and sediment traps (147 and 540 m) for bulk *Pseudo-nitzschia*, total domoic acid (tDA), particulate DA (pDA), dissolved DA (dDA), and cellular DA (cDA). For the Pier Stations, bulk *Pseudo-nitzschia* abundance was further classified as narrow (<3 µm) or wide (>3 µm). Data that are below detection are denoted by BD (200 cells L^−1^ for *Pseudo-nitzschia* abundance or 1.3 ng L^−1^ for DA concentrations). All data are available in Tables S2–S4.

Location	Parameter	N	Min.	Max.	Median	Mean	Std. Dev.
**Pier Stations**	*Pseudo-nitzschia* (cells L^−1^)	465	BD	8.03 × 10^5^	7.15 × 10^3^	4.08 × 10^4^	8.97 × 10^4^
	Narrow (cells L^−1^)	463	BD	7.86 × 10^5^	1.86 × 10^3^	2.23 × 10^4^	7.57 × 10^4^
	Wide (cells L^−1^)	463	BD	3.77 × 10^5^	928	1.86 × 10^4^	4.74 × 10^4^
	*Pseudo-nitzschia* pDA (ng L^−1^)	193	BD	9.33 × 10^3^	94	409	1.04 × 10^3^
	*Pseudo-nitzschia* cDA (pg cell^−1^)	182	BD	833	3.76	17.6	65.2
	Narrow DA (pg cell^−1^)	144	BD	2.14 × 10^3^	4.97	92.7	274
	Wide DA (pg cell^−1^)	149	BD	833	10.77	26.5	72.7
**Surface**	*Pseudo-nitzschia* (cells L^−1^)	223	BD	3.56 × 10^6^	6.66 × 10^4^	2.45 × 10^5^	4.94 × 10^5^
**Stations (1–7)**	*Pseudo-nitzschia* % of Total	223	BD	91.5%	16.5%	25.7%	25.3%
	Total Microphytoplankton (cells L^−1^)	223	5.64 × 10^3^	6.75 × 10^6^	4.16 × 10^5^	8.00 × 10^5^	1.04 × 10^6^
	Chl *a* (ng L^−1^)	251	83.4	2.76 × 10^4^	1.87 × 10^3^	2.90 × 10^3^	3.31 × 10^3^
	tDA (ng L^−1^)	235	BD	2.23 × 10^5^	762	7.82 × 10^3^	2.22 × 10^4^
	pDA (ng L^−1^)	224	BD	1.10 × 10^5^	257	3.65 × 10^3^	1.07 × 10^4^
	dDA (ng L^−1^)	233	BD	1.13 × 10^5^	276	4.38 × 10^3^	1.21 × 10^4^
	cDA (pg cell^−1^)	203	BD	1.44 × 10^3^	1.86	37	124
**Station 4**	*Pseudo-nitzschia* (cells L^−1^)	178	BD	3.83 × 10^6^	2.08 × 10^4^	1.48 × 10^5^	10^5^
**(0–150 m)**	*Pseudo-nitzschia* % of Total	177	BD	98.1%	10.1%	21.5%	26.0%
	Total Microphytoplankton (cells L^−1^)	177	3.60 × 10^3^	5.11 × 10^6^	2.14 × 10^5^	5.07 × 10^5^	8.37 × 10^5^
	Chl *a* (ng L^−1^)	187	20	1.18 × 10^4^	1.35 × 10^3^	2.22 × 10^3^	2.38 × 10^3^
	tDA (ng L^−1^)	243	BD	4.27 × 10^4^	71.46	2.69 × 10^3^	6.81 × 10^3^
	pDA (ng L^−1^)	234	BD	2.18 × 10^4^	19.32	1.17 × 10^3^	3.12 × 10^3^
	dDA (ng L^−1^)	242	BD	2.61 × 10^4^	1.04	1.58 × 10^3^	4.02 × 10^3^
	cDA(pg cell^−1^)	159	BD	323	1.13	18.3	45.6
**147 m Trap**	*Pseudo-nitzschia* (cells g sed.^−1^).	31	1.17 × 10^3^	1.03 × 10^8^	1.74 × 10^5^	1.21 × 10^7^	2.26 × 10^7^
	*Pseudo-nitzschia* flux (cells m^−2^ d^−1^)	31	523	6.00 × 10^7^	9.49 × 10^4^	5.37 × 10^6^	1.31 × 10^7^
	DA concentration (µg g sed.^−1^)	46	BD	79.9	11.6	8.4	17.3
	DA flux (µg m^−2^ d^−1^)	46	BD	70.8	0.7	5.6	13.0
**509 m Trap**	*Pseudo-nitzschia* (cells g sed.^−1^).	22	8.22 × 10^3^	9.54 × 10^6^	4.31 × 10^5^	2.04 × 10^6^	2.64 × 10^6^
	*Pseudo-nitzschia* flux (cells m^−2^ d^−1^)	22	7.59 × 10^3^	1.48 × 10^7^	8.74 × 10^5^	3.07 × 10^6^	4.13 × 10^6^
	DA concentration (µg g sed.^−1^)	28	BD	58.4	1.4	8.0	15.0
	DA flux (µg m^−2^ d^−1^)	28	BD	139.6	2.6	15.2	30.1

## Supplementary Materials

The following are available online at http://www.mdpi.com/2072-6651/10/11/480/s1, Table S1: CTD data; Table S2: Data from SBB Piers; Table S3: Station data; Tables S4: Sediment Trap data.
